# Digital eye health and behavioral determinants of screen use among university students in the UAE

**DOI:** 10.3389/fdgth.2026.1719475

**Published:** 2026-02-12

**Authors:** Mohamed Anas Patni, Wafeeqa Fatima, Maitha Abdulla Alshamsi, Hawa Ali Jama, Mohammad Shafeeq, Rikhil Rajiv, Mouhamad Soubhi Alsoued, Biji Thomas George, Rasha Aziz Attia Salama, Abdalla Ahmed Eldaw Elamin, Thilakavathy Pandurangan, Shadha Nasser Bahutair, Hafiz Ahmad

**Affiliations:** 1 Department of Community Medicine, Ras Al Khaimah Medical and Health Science University, Ras Al Khaimah, United Arab Emirates; 2 Research Unit, Centre for Health Work Force Development, RAKMHSU, Ras Al Khaimah, United Arab Emirates; 3 Department of General Surgery, RAK College of Medical Sciences, RAKMHSU, Ras Al Khaimah, United Arab Emirates; 4 Department of Community Medicine, Kasr El Aini Faculty of Medicine, Cairo University, Cairo, Egypt; 5 Department of Anatomy, RAK College of Medical Sciences, RAKMHSU, Ras Al Khaimah, United Arab Emirates; 6 Department of Microbiology, RAK College of Medical Sciences, RAKMHSU, Ras Al Khaimah, United Arab Emirates; 7 Department of Obstetrics and Gynaecology, RAK College of Medical Sciences, RAKMHSU, Ras Al Khaimah, United Arab Emirates

**Keywords:** behavioral determinants, blue light filter, digital eye strain, digital wellbeing, screen time, UAE, university students

## Abstract

**Background:**

The growing integration of digital technologies into daily life has heightened concerns over visual health, particularly among young adults with prolonged screen exposure, with digital eye strain (DES), dry eye disease (DED), and myopia becoming increasingly prevalent. In the United Arab Emirates (UAE), evidence on screen use and ocular health among university students remains limited. This study assessed the prevalence of eye-related disorders, behavioral correlates of screen use, and preventive practices among university students in the United Arab Emirates (UAE), with implications for digital health and wellbeing interventions.

**Methods:**

A cross-sectional study was conducted among 463 undergraduate students from three universities in Ras Al Khaimah, UAE. A validated self-administered questionnaire assessed demographics, device use, symptoms, and preventive practices (Cronbach's *α* = 0.78). Data were analyzed using descriptive statistics, chi-square tests, *t*-tests, ANOVA, and logistic regression.

**Results:**

Overall, 35.4% of students reported a diagnosed eye disorder. The majority used digital devices for 4–6 h (43.2%) or 7–9 h (34.1%) daily, with smartphones being the most common. Frequent symptoms included headaches (43.8%), neck/back pain (38.2%), eye strain (37.6%), and dry eyes (37.1%). Symptom scores were higher among females (*p* < 0.001) and those with ≥10 h of daily screen time (*p* < 0.001). Logistic regression showed female gender (OR = 1.77), lack of blue light filter use (OR = 0.54), and infrequent breaks (*p* = 0.013–0.037) as significant predictors.

**Conclusions:**

Eye disorders and digital eye strain are prevalent among university students, reflecting behavioral patterns of prolonged and unregulated screen use.

## 1 Introduction

In recent years, the exponential rise in digital screen usage has dramatically reshaped how individuals across all age groups engage with the world, bringing with it a growing concern over the implications for visual health. The proliferation of smartphones, tablets, and computers, especially in the wake of remote work and online education, has been linked to a range of eye-related disorders, including digital eye strain (DES), dry eye disease (DED), and the early onset and progression of myopia (1–4). Studies suggest that digital eye strain may affect over 50% of users, with symptoms such as blurred vision, headaches, and dry eyes becoming increasingly common (2, 5). In particular, children and young adults appear to be especially vulnerable to these visual issues due to extended and unregulated screen exposure (3, 6).

Meta-analyses among children and young adults, largely from Asian and Western populations, indicate that higher digital screen exposure, especially smartphone and tablet use, is associated with a small to moderate increase in myopia risk, with statistically significant but heterogeneous effect sizes influenced by duration of exposure and study setting (3, 7). Concurrently, digital screen exposure has also been identified as a strong external risk factor for dry eye disease, with studies reporting worsened ocular metrics, such as reduced blink rates and tear film instability among heavy screen users (8, 9). Several interventions have been proposed to reduce digital eye strain, including blue-light filtering lenses and scheduled visual breaks. While some experimental and short-term studies report improvements in subjective symptoms such as glare, visual discomfort, and eye fatigue, systematic reviews and randomized trials show inconsistent or minimal benefits on objective visual outcomes, with effects often comparable to placebo or standard ergonomic advice. These mixed findings contribute to the inconclusive evidence regarding the overall effectiveness of current interventions (2, 4, 10).

Globally, the increasing burden of digital eye strain has prompted the inclusion of screen-related eye health within broader digital wellbeing and occupational health discussions. In high-income and digitally advanced countries, the absence of local epidemiological data may delay recognition of emerging health risks and limit the development of targeted preventive strategies (2). In the United Arab Emirates (UAE), digital device use is exceptionally high, reflecting widespread smartphone ownership, high internet penetration, and the rapid adoption of digital learning platforms. National and international reports indicate that over 98% of the UAE population has internet access and smartphone ownership exceeds 90%, with young adults and university students representing the most intensive users (11). Despite this, local evidence on the prevalence and determinants of digital eye-related disorders remains limited and largely confined to single-institution studies (12). A recent study among medical students in the UAE reported that 92.8% experienced at least one symptom of digital eye strain, with nearly three-quarters using digital devices for more than three hours daily and almost one-third exceeding six hours per day. While these findings highlight a substantial symptom burden, most available studies are restricted to single institutions and rely predominantly on self-reported symptoms, limiting their utility for informing institutional health policies and national digital health strategies (12, 13).

Given the increasing reliance on digital technologies in higher education, prolonged unregulated screen exposure among students may contribute to worsening visual discomfort, reduced academic productivity, and long-term ocular health consequences. Addressing these gaps through systematically collected local evidence is therefore essential to guide preventive interventions, ergonomic guidelines, and digital wellbeing initiatives within university settings in the UAE. This study aims to explore the prevalence of eye-related disorders and their correlation with digital device use among students and young adults. The findings intend to inform both public health awareness and institutional interventions tailored to the region's specific digital habits and healthcare needs.

## 2 Materials and methods

### 2.1 Study design

This university-based cross-sectional study was conducted among undergraduate students enrolled at three higher education institutions in Ras Al Khaimah, United Arab Emirates (UAE): Ras Al Khaimah Medical and Health Sciences University (RAKMHSU), the American University of Ras Al Khaimah (AURAK), and the Higher Colleges of Technology (HCT), Ras Al Khaimah campus. These institutions were selected to capture a diverse yet comparable student population, representing medical and health sciences, engineering, business, and applied sciences disciplines within the same geographic and sociocultural setting. All three universities operate under similar national higher education regulations, follow English-medium instruction, and cater to a predominantly young adult population with high exposure to digital learning platforms, particularly following the expansion of blended and online education models in the UAE.

### 2.2 Study population and sample size

The study population comprised undergraduate students aged 17 years and above enrolled at RAKMHSU, AURAK, and HCT during the study period between December 2024 and May 2025. These universities collectively represent a substantial proportion of the undergraduate student population in Ras Al Khaimah, with estimated enrollments of approximately 1,600 students at RAKMHSU, 800 students at AURAK, and 600 students at HCT (RAK campus). A minimum sample size of 384 participants was calculated using OpenEpi software, assuming a 50% expected prevalence (to maximize sample size), 95% confidence level, and 5% margin of error, appropriate for prevalence estimation in cross-sectional studies where local estimates are limited. Convenience sampling was employed due to feasibility constraints and voluntary participation. A total of 463 eligible students were ultimately recruited, exceeding the minimum required sample size and thereby improving the precision and stability of the estimates.

Participants with severe pre-existing ocular conditions such as retinal detachment were excluded because their symptoms and visual impairment are driven by underlying pathology and clinical management rather than digital device exposure. Inclusion of such cases could confound symptom attribution and bias associations between screen-use behaviors and eye-related outcomes.

Participant recruitment was feasibility-based and conducted using both paper-based and online questionnaires across all three universities. As the investigators were institutionally based at Ras Al Khaimah Medical and Health Sciences University (RAKMHSU), direct on-site access facilitated higher participation at this institution. Although paper-based and online recruitment were also attempted at the American University of Ras Al Khaimah and the Higher Colleges of Technology, response rates at these institutions were comparatively lower.

### 2.3 Data collection tool and procedure

Data were collected using a questionnaire, that was self-developed for this study based on a review of previously published literature on digital eye strain, computer vision syndrome, dry eye symptoms, and screen-use behaviors. Items were adapted from commonly reported symptom lists and behavioral measures used in prior studies, rather than from a single validated digital eye strain or dry eye disease instrument. The questionnaire comprised four sections, predominantly using closed-ended, categorical response options.

A) The demographic section included items on age, gender, nationality, university, academic level and year, field of study, and use of corrective lenses.
B) Digital device use was assessed through multiple-choice questions capturing average daily screen time (categorized into five duration bands), primary device type, purpose of use, duration of continuous use without breaks, frequency of breaks, and use of blue-light filters or glasses.
C) Eye-related symptoms were assessed by asking participants to indicate the presence of common symptoms experienced after prolonged digital device use, including eye strain, blurred vision, dry eyes, myokymia, headaches, neck/back pain, and light sensitivity. Responses were recorded using binary (yes/no) options. A total symptom score was calculated by summing the number of symptoms reported, yielding a score range from 0 to 7, with higher scores indicating greater symptom burden. Additional items captured symptom frequency, self-reported diagnosis of eye disorders, and types of diagnosed conditions. The symptom score reflects overall self-reported symptom burden and was not intended to diagnose digital eye strain or dry eye disease.
D) The impact and awareness sections assessed perceived effects of digital device use on vision, adherence to eye-strain prevention practices, behavioral changes related to eye discomfort, perceived academic impact, awareness of long-term risks, and perceived adequacy of university-level eye health awareness initiatives.

The questionnaire underwent content review by subject experts and pilot testing among approximately 30 students to assess clarity, comprehension, and completion time. Minor wording refinements were made based on feedback, and pilot responses were excluded from the final analysis. Internal consistency of the symptom-related items demonstrated acceptable reliability (Cronbach's *α* = 0.78). No formal construct validation was performed, which is acknowledged as a limitation. The questionnaire was both online (through Google Forms) and available in hard copy for convenience. The answers were coded numerically and analyzed. To minimize duplicate responses in the online survey, Google Forms settings were configured to restrict submissions to one response per account where applicable, and participants were instructed to complete the questionnaire only once. During data cleaning, responses were screened for potential duplication by examining identical demographic profiles and response patterns. Paper-based questionnaires were collected in person, ensuring that each paper form represented a single unique participant.

### 2.4 Ethical issues

The study was conducted in accordance with the Declaration of Helsinki, and approved by the Institutional Review Board of Ras Al Khaimah emirates. (Approval No: MOHAP/REC/2022/19-2023-UG-M). Participation in the research was voluntary, and informed consent was obtained from all the participants before data collection.

### 2.5 Data analysis

The collected data was entered into Google Sheets and analyzed using IBM SPSS version 29. Descriptive statistics were used to calculate frequencies, means, and standard deviations. The total symptom score demonstrated an approximately normal distribution on visual inspection and normality testing; however, as it represents a discrete count variable, both parametric and non-parametric summary statistics were reported to aid interpretability. For categorical comparisons with sparse cell counts, results were interpreted cautiously, and Fisher's exact test was applied where chi-square assumptions were not met. Mean total symptom scores were compared between two independent groups (e.g., male vs. female; Emirati vs. non-Emirati) using an unpaired (independent-samples) *t*-test. Comparisons across variables with more than two categories (e.g., university and academic year) were performed using one-way ANOVA. For logistic regression analyses, standard assumptions were evaluated. Multicollinearity among independent variables was assessed using the variance inflation factor (VIF), with all VIF values below commonly accepted thresholds, indicating no significant multicollinearity. Model fit was evaluated using the Hosmer–Lemeshow goodness-of-fit test, which indicated adequate model calibration. Regression results were therefore considered statistically valid. Categorical variables were coded using reference categories selected *a priori* based on conceptual relevance (e.g., male sex, non-use of blue-light filters, and regular break-taking as reference groups). Variables were included in multivariable logistic regression models based on theoretical relevance and prior evidence linking screen-use behaviors to eye-related outcomes, rather than solely on univariate significance. Statistical significance was at *p* < 0.05.

## 3 Results

A total of 463 participants participated in the study. The majority of participants were female (*n* = 300, 64.8%), while males accounted for 35.2% (*n* = 163). Most participants were aged 17–20 years (57.2%), followed by 21–23 years (39.7%), 24–26 years (1.9%), and 27 years or older (1.1%). The majority of them were non-Emirati (83.4%). Most participants were from RAKMHSU (77.8%), followed by AURAK (18.4%) and HCT (3.9%). Bachelor's degree students constituted the majority of the study participants (97.4%), and most of the participants were enrolled in medicine and health sciences (78.2%). Overall, 164 participants (35.4%) reported having at least one diagnosed eye disorder.

Out of 463 participants, 243 (52.4%) participants reported using corrective lenses (glasses and/or contact lenses). Most participants used digital devices for 4–6 h (*n* = 200) or 7–9 h (*n* = 158). In contrast, extended use beyond 10 h was less frequent (*n* = 43), and only a few reported minimal use of less than 1 h (*n* = 7). Smartphones were the most commonly used devices (*n* = 303), followed by tablets (*n* = 79) and laptops (*n* = 77). Participants primarily used digital devices for academic tasks (*n* = 170) and social media (*n* = 178). In terms of usage habits, 221 participants spend more than 3 consecutive hours on digital devices without taking breaks, while 242 reported not doing so. Break frequency varied among students, with the majority taking regular breaks every 30 min (*n* = 144) or every hour (*n* = 157). As for eye protection, 199 participants reported using blue light filters or glasses, while the majority (*n* = 264) did not use any form of protection (Table 1).

**Table 1:** Digital device usage patterns among study participants.

Variable	Category	Frequency (%)
Use of corrective lens	No	220 (47.5%)
	Yes, contact lenses	6 (1.3%)
	Yes, glasses	179 (38.7%)
	Yes, both	58 (12.5%)
Daily screen time	<1 h	7 (1.5%)
	1–3 h	55 (11.9%)
	4–6 h	200 (43.2%)
	7–9 h	158 (34.1%)
	>10 h	43 (9.3%)
Most frequently used digital device	Laptop/PC	77 (16.6%)
	Smartphone	303 (65.4%)
	Tablet	79 (17.1%)
	Other	4 (0.9%)
Primary purpose of use	Academic	170 (36.7%)
	Entertainment	110 (23.8%)
	Social media	178 (38.4%)
	Other	5 (1.1%)
Continuous use >3 h without break	Yes	221 (47.7%)
	No	242 (52.3%)
Break frequency	Every 30 min	144 (31.6%)
	Every 1 h	157 (34.5%)
	Every 2–3 h	95 (20.9%)
	Rarely	59 (13.0%)
Blue light filter/glass use	Yes	199 (43.0%)
	No	264 (57.0%)

**Figure 1:**
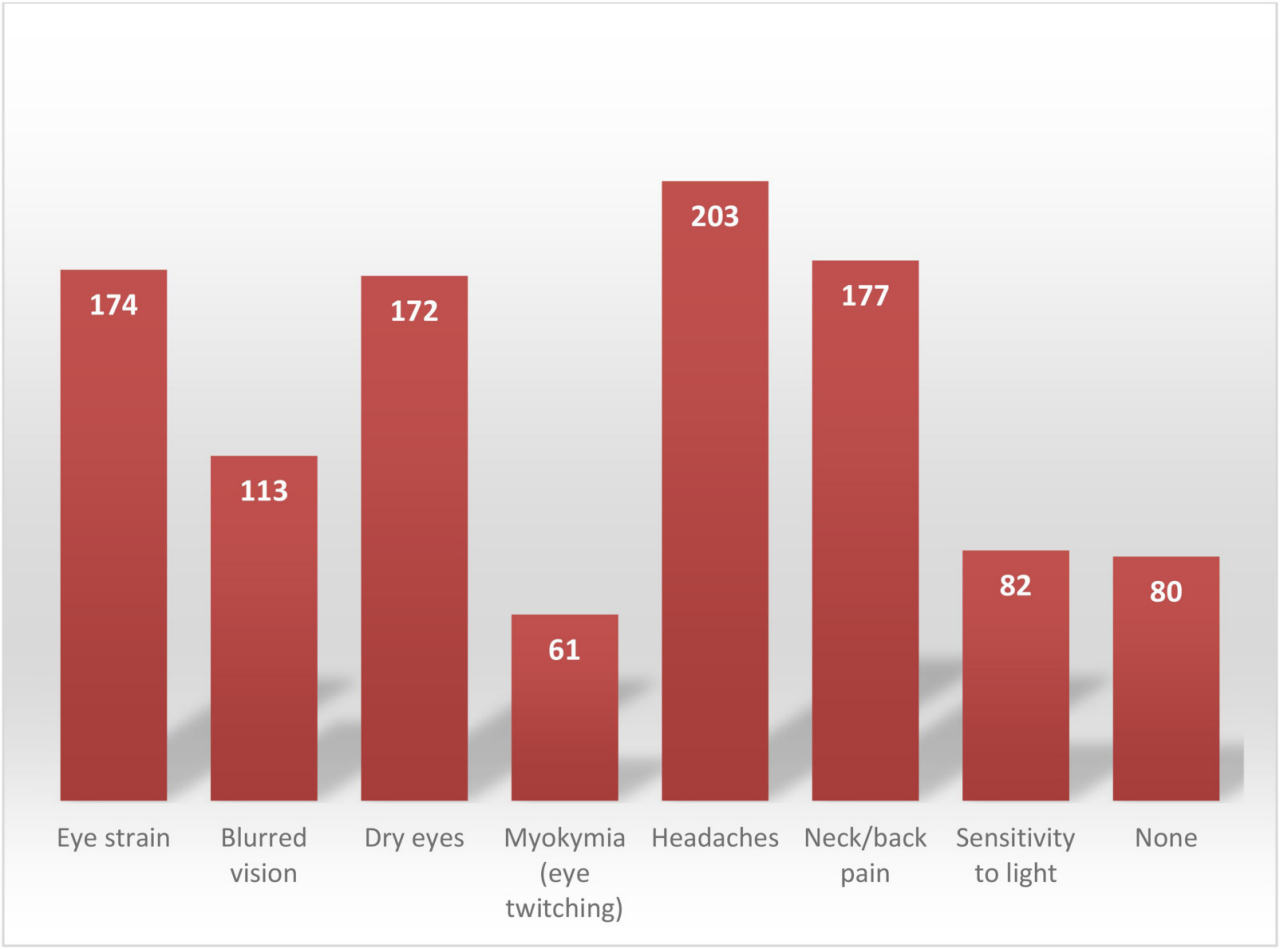
Reported frequencies of specific Eye-related symptoms.

Figure 1 illustrates the distribution of individual eye-related symptoms reported by participants. Overall, headache, neck/back pain, eye strain, and dry eyes were the most frequently reported symptoms, whereas myokymia and light sensitivity were less commonly reported.

The total symptom score was calculated for each student by counting how many different eye-related symptoms they reported. The average symptom score was 2.12 ± 1.62, with a median of 2, minimum of 0, and maximum of 7. The interquartile range (IQR) was 1–3, indicating that 50% of students experienced between 1 and 3 symptoms with a minority reporting experiencing 5 or more.

The relationship between symptom scores across different sociodemographic groups is detailed in Table 2. Females had higher mean symptom scores than males (2.35 ± 0.31vs 1.69 ± 0.31, *p* < 0.001), suggesting that female students may experience greater eye-related discomfort. However, when comparing different university affiliations (AURAK, HCT, RAKMSHU) and academic year (1st to 6th), it was found that there was no significant association in their groups (*p* = 0.871 and *p* = 0.163, respectively). Furthermore, no significant differences were found between Emirati and non-Emirati students (*p* = 0.186). Academic year and nationality did not show any meaningful associations with symptoms severity as well.

**Table 2:** Mean total symptom score by sociodemographic characteristics.

Category	Group	Mean score ± SD	Test type	Test statistic	*p*-value
Gender	Male	1.69 ± 0.31	Unpaired *t*-test	4.246	<0.001*
	Female	2.35 ± 0.36			
Nationality	Emirati	2.35 ± 0.35	Unpaired *t*-test	1.332	0.1858
	Non-Emirati	2.08 ± 0.32			
University	AURAK	2.16 ± 0.33	One way ANOVA	0.138	0.8709
	HCT	2.28 ± 0.3			
	RAKMHSU	2.1 ± 0.32			
Academic year	1st	1.95 ± 0.28	One way ANOVA	1.584	0.1631
	2nd	2.2 ± 0.34			
	3rd	2.36 ± 0.35			
	4th	1.79 ± 0.27			
	5th	2.18 ± 0.33			
	6th	3.0 ± 0.38			

* Statistically significant results at *p* < 0.05.

The relationship between symptom scores in accordance to digital device usage is detailed in Table 3. Screen time duration was significant in its correlation with mean symptom score (*p* < 0.001), with the highest mean scores seen in patients with 10+ h of screen time compared to those who used less than 1 h of screen time. Participants who reported using blue light filter had higher mean scores in comparison to those who did not use the filter, this finding was significant (*p* = 0.0427).

**Table 3:** Mean total symptom score by digital device usage.

Category	Group	Mean score ± SD	Test type	Test statistic	*P*-value
Screen time	10+ h	2.98 ± 0.31	One way ANOVA	253.6	<0.001*
	1–3 h	2.09 ± 0.21			
	4–6 h	1.99 ± 0.22			
	7–9 h	2.16 ± 0.19			
	<1 h	0.75 ± 0.18			
Break frequency	Every 1 h	2.014 ± 0.27	One way ANOVA	2.73	0.1343
	Every 2–3 h	2.31 ± 0.32			
	Every 30 min	2.05 ± 0.32			
	Never	2.07 ± 0.21			
	Rarely	2.513 ± 0.23			
Blue light filter use	No	2.04 ± 0.26	Unpaired *t*-test	8.36	0.0046*
	Yes	2.24 ± 0.25			

* Statistically significant results at *p* < 0.05.

Table 4 summarises the analysis of the association between various demographic factors and the prevalence of diagnosed eye disorders among university participants. The age distribution among those diagnosed was relatively even, with no statistically significant association observed between age groups and diagnosis rates (*p* = 0.4705). Notably, the amount of time spent using digital devices consecutively, whether three or more hours without a break, did not demonstrate a statistically significant relationship with eye disorder diagnosis (*p* = 0.6397).

**Table 4:** Cross tabulations of demographics with diagnosed eye disease.

Variable	Category	Diagnosed: Yes	Diagnosed: No	Chi-square	*p*-value
Age	17–20	88	177	2.53	0.4705
	21–23	72	111		
	24–26	2	7		
	27+	2	3		
Consecutive use >3 h	No	83	159	0.22	0.6397
	Yes	81	139		
Blue light filter/Glasses use	No	63	200	34.37	<0.001*
	Yes	101	98		
Field of study	Business/Management	5	10	7.27	0.0637
	Engineering	4	26		
	Medicine/Health Sciences	136	225		
Gender	Female	118	181	5.34	0.0208*
	Male	46	117		
Daily screen time	1–3 h	18	37	3.08	0.5437
	10+ h	16	27		
	4–6 h	64	135		
	7–9 h	64	94		
Break frequency	Every 1 h	57	100	4.47	0.3459
	Every 2–3 h	30	65		
	Every 30 min	58	86		
	Never	1	7		
	Rarely	18	40		
Nationality	Emirati	24	53	0.55	0.4598
	Non-Emirati	140	245		
University	AURAK	23	62	3.38	0.1848
	HCT	6	12		
	RAKMHSU	135	224		
Most used digital device	Laptop/PC	27	50	3.8	0.2839
	Other	2	2		
	Smartphone	100	202		
	Tablet	35	44		

* Statistically significant results at *p* < 0.05.

Similarly, most participants reported using either smartphones or laptops as their main digital devices. However, the kind of device participants used didn't seem to significantly affect their chances of being diagnosed with an eye condition (*p* = 0.2839).

Interestingly, a marked difference was observed with respect to the use of blue light filters or glasses; participants who did not use blue light filters had a significantly higher rate of diagnosed eye disorders (*p* < 0.001), highlighting a potentially protective effect of such interventions. The study sample showed a clear gender imbalance, with female participants not only outnumbering males but also being more likely to receive a diagnosis of eye disorders compared to their male peers (*p* = 0.0208). No statistically significant differences were found regarding the hours per day spent on digital devices (*p* = 0.5437), frequency of breaks taken while using devices (*p* = 0.3459), nationality (*p* = 0.4598), field of study (*p* = 0.0637), or university attended (*p* = 0.1848), suggesting that these factors did not independently influence the likelihood of receiving an eye disorder diagnosis within this cohort.

Table 5 summarises the logistic regression analysis, which offers a clearer picture of the key factors that significantly predict eye disorder diagnoses. In the multivariable logistic regression analysis, female students had significantly higher odds of reporting a diagnosed eye disorder compared with males (OR = 1.77, 95% CI: 1.22–2.63, *p* = 0.037). Students who rarely took breaks during digital device use also demonstrated increased odds of diagnosed eye disorders relative to those taking breaks every 30 min (OR = 1.47, 95% CI: 1.12–1.71, *p* = 0.037). Conversely, use of blue-light filters or glasses was associated with significantly lower odds of diagnosed eye disorders (OR = 0.54, 95% CI: 0.36–0.79, *p* = 0.014). Nationality, field of study, daily screen time, and age group were not significantly associated with diagnosed eye disorders after adjustment.

**Table 5:** Multivariable logistic regression analysis of factors associated with diagnosed eye disorders.

Variable	Comparison	Odds ratio [Exp(B)]	95% CI	*p*-value
Gender	Female vs. Male	1.77	1.22–2.63	0.037*
Blue light filter use	No vs. Yes	0.54	0.36–0.79	0.014*
Break frequency	Rarely vs. every 30 min	1.47	1.12–1.71	0.037*
Nationality	Emirati vs. non-Emirati	1.21	0.83–1.77	0.32
Field of study	Non-health vs. Health sciences	0.94	0.65–1.36	0.74
Daily screen time	≥7–9 h vs. 1–3 h	1.19	0.84–1.69	0.33
	≥10 h vs. 1–3 h	1.41	0.89–2.25	0.14
Age group	21–23 vs. 17–20	1.12	0.82–1.54	0.46
	≥24 vs. 17–20	1.38	0.71–2.66	0.34
Primary device	Smartphone vs. others	1.11	0.77–1.60	0.57

* Statistically significant results at *p* < 0.05.

## 4 Discussion

This study provides multi-university evidence on the burden of digital eye-related symptoms and associated behavioral determinants among university students in the UAE. Rather than merely confirming the high prevalence of symptoms reported in earlier single-institution studies, the findings extend existing knowledge by identifying modifiable behavioral factors, such as prolonged uninterrupted screen use, infrequent breaks, and limited use of protective measures, that are associated with both symptom burden and diagnosed eye disorders in a young adult population.

The high frequency of symptoms such as headache, eye strain, dry eyes, and neck/back pain observed in this study is consistent with the recognized clinical profile of digital eye strain (DES) (2, 5). Prolonged digital device use is known to reduce blink rate and increase blink incompleteness, leading to tear film instability and ocular surface dryness, which may explain the prominence of dry eye symptoms in this cohort (8, 9). Sustained near-work activity without adequate breaks can also increase accommodative demand and visual fatigue, contributing to eye strain and headache (2, 6). Musculoskeletal symptoms, particularly neck and back pain, likely reflect suboptimal ergonomics during extended screen use, which has been reported in both student and occupational populations (14, 15).

The higher symptom burden and increased odds of diagnosed eye disorders among female participants observed in this study may be explained by a combination of biological and behavioral factors. Females have been shown to be more susceptible to dry eye disease due to hormonal influences affecting tear film composition and ocular surface homeostasis (16, 17). Additionally, previous studies suggest that female students may engage in longer cumulative screen exposure through academic and social activities, potentially amplifying symptom risk (18, 19). These findings reinforce growing evidence that gender-sensitive approaches may be necessary when designing preventive strategies for digital eye health.

Although total daily screen time was not independently associated with diagnosed eye disorders in multivariable analysis, behavioral patterns such as infrequent breaks and lack of blue-light filter use emerged as significant predictors. This suggests that how screens are used may be more important than how long they are used. Experimental studies have demonstrated that regular visual breaks, such as adherence to the 20-20-20 rule, can reduce subjective symptoms of eye fatigue and dryness, even when total screen exposure remains high (10, 20). The protective association observed with blue-light filter use should be interpreted cautiously; while some short-term studies report reduced glare and visual discomfort, high-quality systematic reviews have found inconsistent or minimal benefits on objective visual outcomes (4, 21). This finding may reflect reverse causality or health-seeking behaviour, whereby individuals experiencing greater visual discomfort are more likely to adopt protective strategies such as blue-light filters, potentially reducing longer-term risk of clinical diagnosis without immediately alleviating subjective symptoms.

From a clinical perspective, the findings suggest that digital eye strain and related symptoms are common even among young adults without advanced ocular pathology. This highlights the importance of incorporating brief screening for digital eye strain symptoms into routine optometry and ophthalmology consultations, particularly for students and young professionals. Simple behavioral counselling, focused on regular breaks, ergonomic positioning, and screen-use habits, may help mitigate symptom progression and reduce unnecessary visual discomfort (6, 22).

At a public health level, the high symptom burden identified in this study underscores the need to integrate digital eye health into broader student wellbeing and digital health initiatives. Universities represent a critical intervention setting, as students are exposed to prolonged screen use through digital learning platforms and academic requirements. Institutional strategies such as awareness campaigns, ergonomic training, promotion of regular visual breaks, and inclusion of digital wellbeing modules in student orientation programs may offer cost-effective approaches to reducing symptom burden (13, 23).

At the policy level, the findings highlight an important gap in the integration of digital eye health within national digital transformation and occupational health frameworks. In digitally advanced settings such as the UAE, where internet penetration and digital learning adoption are among the highest globally, evidence-based guidelines addressing safe screen use in educational environments are increasingly necessary. The results of this study support the inclusion of digital eye strain prevention within national digital health strategies, aligning technological advancement with sustainable health outcomes (24, 25).

A key strength of this study is the inclusion of students from multiple universities within the same emirate, providing broader contextual insight than prior single-institution studies. The assessment of both behavioral determinants and self-reported diagnoses further enhances the interpretability of the findings. However, several limitations should be acknowledged. The questionnaire was self-developed and, although informed by existing literature and reviewed for content clarity, was not based on a fully validated digital eye strain or dry eye disease instrument. The total symptom score reflects overall symptom burden rather than a clinical diagnosis of digital eye strain or dry eye disease. The use of self-reported data may introduce recall bias. Convenience sampling was used due to feasibility constraints, and the sample was dominated by students from medical and health science programs, which may limit generalizability to the broader university student population. Despite these limitations, the study provides valuable multi-institutional evidence on modifiable behavioral factors related to digital device use among university students in the UAE.

Future research should consider longitudinal and interventional designs to evaluate the effectiveness of targeted behavioral interventions, such as structured break reminders or ergonomic training, in reducing digital eye strain among students. Incorporating objective clinical measures alongside self-reported symptoms may further strengthen the evidence base and support policy translation.

## 5 Conclusion

Eye-related disorders are common among university students in the UAE and are strongly associated with digital behavior patterns such as prolonged screen exposure, inadequate blue-light protection, and infrequent visual breaks. These modifiable risk factors can be mitigated through targeted digital health communication and behavioral interventions.

Incorporating digital wellbeing tools, such as app-based screen-time reminders, adaptive lighting technologies, and campus-wide awareness initiatives can foster healthier technology habits. Future digital health programs should prioritize user-centered design and behavioral personalization to encourage long-term adherence to preventive practices.

By framing digital eye strain within the broader context of digital wellbeing and health communication, this study connects ophthalmologic insights with digital health innovation, providing actionable strategies to preserve visual health in increasingly connected academic environments.

## Data Availability

The datasets generated and/or analyzed during the current study are available from the corresponding author upon reasonable request.
